# Evaluation of Radiomics to Predict the Accuracy of Markerless Motion Tracking of Lung Tumors: A Preliminary Study

**DOI:** 10.3389/fonc.2018.00292

**Published:** 2018-07-31

**Authors:** Kevin Nguyen, Maksat Haytmyradov, Hassan Mostafavi, Rakesh Patel, Murat Surucu, Alec Block, Matthew M. Harkenrider, John C. Roeske

**Affiliations:** ^1^Stritch School of Medicine, Loyola University Chicago, Maywood, IL, United States; ^2^Department of Radiation Oncology, Loyola University Medical Center, Maywood, IL, United States; ^3^Varian, Palo Alto, CA, United States

**Keywords:** lung cancer, motion tracking, dual energy imaging, radiomics, template matching

## Abstract

Template-based matching algorithms are currently being considered for markerless motion tracking of lung tumors. These algorithms use tumor templates derived from the planning CT scan, and track the motion of the tumor on single energy fluoroscopic images obtained at the time of treatment. In cases where bone may obstruct the view of the tumor, dual energy fluoroscopy may be used to enhance soft tissue contrast. The goal of this study is to predict which tumors will have a high degree of accuracy for markerless motion tracking based on radiomic features obtained from the planning CT scan, using peak-to-sidelobe ratio (PSR) as a surrogate of tracking accuracy. In this study, CT imaging data of 8 lung cancer patients were obtained and analyzed through the open source IBEX program to generate 2,287 radiomic features. Agglomerative hierarchical clustering was used to narrow down these features into 145 clusters comprised of the highest correlation to PSR. The features among the clusters with the least inter-correlation were then chosen to limit redundancy in the data. The results of this study demonstrated a number of radiomic features that are positively correlated to PSR. The features with the highest degree of correlation included complexity, orientation and range. This approach may be used to determine patients for whom markerless motion tracking would be beneficial.

## Introduction

It is estimated that nearly 190,000 new cases of non-small cell lung cancer (NSCLC) are diagnosed in the United States each year, accounting for ~130,000 deaths annually (1). Stereotactic body radiation therapy (SBRT) is a highly effective treatment method for early stage NSCLC patients who are medically unfit for lobectomy (2). SBRT allows for the administration of high doses of radiation to the tumor and has several advantages over conventional treatment. Moreover, it requires far fewer treatment sessions with improved local control of ≥90% in many studies compared to the 30–60% control rates of conventional external beam radiation therapy (EBRT) (1, 3).

Due to the highly conformal nature of SBRT and high dose per fraction with a limited number of fractions, motion management is critical to ensure that the tumor receives the full dose of radiation and the volume of irradiated normal tissue is minimized. To achieve these endpoints, there has been recent interest in lung tumor tracking during treatment delivery (4–11). The goal of these approaches is to modify the treatment (i.e., multi-leaf collimators, treatment table position, etc.), based on the position of the tumor during respiration to minimize the volume of normal tissue irradiated. This concept is critical in SBRT, as several studies have demonstrated an increased incidence of symptomatic clinical radiation pneumonitis correlating with increased volumes of normal lung irradiated (12–16). While the dosimetric and clinical results appear promising, relying on implanted markers may limit the number of patients who can receive such advanced therapies due to the risks associated with marker implantation. Previous studies with implanted fiducial markers in lung tumors demonstrate this invasive procedure carries the risk of pneumothorax and pulmonary hemorrhage (17). Moreover, migration of fiducial markers in the lung specifically may be significant (4–6).

As an alternative to using implanted markers, several studies have described using fluoroscopy for markerless tumor tracking. These studies have reported target localization <3 mm using fluoroscopy and template matching methods (8–11). However, this method often fails, or results in decreased tracking accuracy, when the boundary of the tumor is obscured by overlying bones. Lewis et al. showed that the tracking error could be up to 5 mm due to the obstruction of the tumor by a high contrast object, such as bone (11). To address this problem, dual energy (DE) imaging has been shown to improve the visualization of tumors and in turn, improve the accuracy of markerless tumor tracking (18). Briefly, DE imaging involves obtaining x-ray images at high (i.e., 120 kVp) and low (i.e., 60 kVp) energies. By performing a weighted-logarithmic subtraction, a third image is created that suppresses bone and enhances soft tissue/tumor.

In a recent DE study by Block et al., 74 image pairs from 17 patients were evaluated using a template matching algorithm (19). The algorithm successfully matched the template in 61 (82%) of the single energy (SE) images and 74 (100%) of the DE images (*p* < 0.01). The mean distance between the gross tumor volume (GTV) centroid coordinates (x,y) of the matched template and physician defined ground truth coordinates was 3.2 ± 2.8 mm for SE vs. 2.3 mm ± 1.7 mm for DE (*p* = 0.03). The false detection rate (fraction of images with >5 mm matching errors) was 7/74 (9.4%) for DE images, vs. 9/74 (12.1%) for SE images (*p* = 0.79). It was also determined that the peak-to-sidelobe ratio (PSR)—a measure of tracking quality—was predictive of template matching accuracy. For PSR <3, the matching accuracy was 3.3 ± 3.1 mm vs. 2.2 ± 1.3 mm for PSR ≥3 (*p* = 0.02). Importantly, the false detection rate was 20.9% (PSR < 3) vs. 4.0% (PSR ≥ 3) (*p* < 0.01). Therefore, the PSR can be used as a predictive surrogate for the accuracy of template matching.

The goal of this study is to determine if one can predict which tumors will have a high degree of accuracy for markerless motion tracking using radiomic features obtained from the planning CT scan. Radiomics is the high throughput extraction and analysis of quantitative features from imaging data (20). These quantitative features have prognostic and predictive potential containing information that cannot be obtained via inspection of the imaging data alone (21, 22). The information found from these features is multidimensional and can comprise of anything from tumor malignancy to predicting patient outcomes (23, 24). To our knowledge, this study represents the first to consider the use of radiomic parameters to predict the success of a markerless motion tracking technique.

## Materials and methods

### Patient selection

In this Loyola University Medical Center Institutional Review Board (IRB) approved study (LU203840), SBRT patients with Stage IA-IIA NSCLC were enrolled in a prospective imaging study. Patients who were eligible for this study included those with no implanted fiducials and a Karnofsky Performance Status (KPS) >70. In total, 8 patients were enrolled and a total of 23 imaging fractions were evaluated.

### CT simulation and treatment planning

Patients were simulated in the supine position, and immobilized using a custom body mold (Alpha Cradle, Smithers Medical Products, Inc., Canton, OH) that was indexed to the treatment table. Simulation was performed using a dedicated CT scanner (Brilliance Large Bore, Philips Medical Systems, Andover, MA) equipped with the Real-Time Position Management (RPM) System (Varian Medical Systems, Palo Alto, CA) to allow for 4D acquisitions. A slice thickness of 3 mm was used for all patients. The tumor volume and prescription were determined by the treating radiation oncologist. Treatment plans, using a volumetric modulated arc therapy (VMAT) technique were created in conjunction with the dosimetry team and ultimately approved by the radiation oncologist.

### Fluoroscopic image acquisition

All patients were treated on a Varian TrueBeam (Varian Medical Systems, Inc., Palo Alto, CA) linear accelerator equipped with an on-board imaging system and RPM. Following each fraction, fluoroscopic images were obtained sequentially using 60 and 120 kVp, respectively. These energies were based on previous studies in which the radiographic technique was optimized for DE subtraction (25, 26). The image sequences were acquired at fixed gantry angles along the same trajectory that was used for VMAT delivery. The Varian TrueBeam Integrated Imaging Solution® in conjunction with the Varian iTools Capture (Varian Medical Systems) software and a Matrox Imaging (Quebec, Canada) frame grabber card were used to acquire a total of 2,448 fluoroscopy frames.

### Dual energy subtraction

The raw images, obtained from the iTools Capture software, were exported off-line into a customized program developed in MATLAB (MathWorks, Natick, MA). Alignment of the individual fluoroscopic frames (60 and 120 kVp) was performed based on the image amplitude and phase as obtained by RPM. Further refinements were made using a localized rigid registration (27). Based on the aligned images, a pixel-by-pixel, weighted logarithmic subtraction was performed to create the DE image using (25, 26):

(1) $$\begin{array}{l} ln\left(I^{DE_{Soft}}\right)=ln\left(I^{High}\right)-w_{s}ln\left(I^{Low}\right) \end{array}$$

where ${\text{I}}_{\text{Soft}}^{\text{DE}}$ is the intensity of the resultant DE soft tissue (bone suppressed) image, I^High^ and I^Low^ are the intensities the high and low energy image pixels, respectively, *ln* is the natural logarthim and w_s_ is the relative weight required to produce the DE image. The 120 kVp images were used as the standard SE images to which the DE images were compared.

### Template matching

Template matching was performed using an algorithm based on Mostafavi et al. (28). First, templates are generated from the CT where the radiation oncologist contours the GTV during routine RT planning. The program then creates a volume of interest (VOI) around the GTV and the voxel values within this volume are forward projected to create templates that are similar to digitally reconstructed radiographs (DRRs). Templates are generated for all expected treatment angles and the appropriate template is selected based on imaging angle (28). Next, the match location is determined by shifting the template across the image and calculating the normalized cross-correlation (NCC) between the template and the acquired image at different 2D offsets within the search region. The NCC is given by (28):

(2) $$\begin{array}{l} NCC=\frac{1}{n}\sum_{x,y}\frac{\left(f\left(x,y\right)-f\right)\left(t\left(x,y\right)-t\right)}{\sigma_{f}\sigma_{t}} \end{array}$$

where *n* is the number of pixels in the template *t*_(x, y)_ and sub-image *f*_(x, y)_, *t* and *f* are the average of the pixel values in the template and sub-image, respectively, while σ_f_ and σ_t_ are the standard deviations over the respective regions. Calculation of NCC at different 2D offsets within a search region results in a match score surface. The offset at which NCC has the maximum value (i.e., the position of the peak of the match score surface) represents the potential target position within the search region. The strength of this peak relative to NCC values away from the peak, called sidelobe values, is quantified by the peak-to-sidelobe ratio (PSR) and is calculated by (28):

(3) $$\begin{array}{l} PSR=\frac{\left[peakvalue-meansidelobe\right]}{\left[sidelobe\;standard\;deviation\right]} \end{array}$$

### Radiomic analysis

For the study, CT imaging data from 8 lung cancer patients treated with SBRT was obtained from Eclipse treatment planning system (Varian Medical Systems, Palo Alto, CA) and analyzed using the open source IBEX program (28). The GTV, as outlined on the 50% respiratory phase CT was the ROI chosen for all of the patients and imaging features of the following feature sets were calculated: Gray Level Run Matrix 25 (*n* = 33 features), Intensity Direct (*n* = 55 features), Intensity Histogram (*n* = 49 features), Neighbor Intensity Difference 3 (*n* = 5 features), Neighbor Intensity Difference 25 (*n* = 5 features), Gray Level Co-occurrence Matrix 3 (*n* = 1792 features), Gray Level Co-occurrence Matrix 25 (*n* = 330 features), and Shape (*n* = 18 features). A summary of these feature sets is presented in Table II of Zhang et al. (29). Of note, the number of features listed here may not be consistent with Zhang et al. (29). The reason is that certain features (such as Percentile) will take on discrete values that are not specifically listed in the table.

A total of 2,287 imaging features were identified in initial dataset for all 8 patients. Imaging features that were undefined or missing for certain patients were removed from the dataset. Additionally, some imaging features from Intensity Direct and Intensity Histogram sets contained duplicate entries, which were subsequently removed. After the pre-processing, the number imaging features decreased to 1,419.

**Figure 1:**
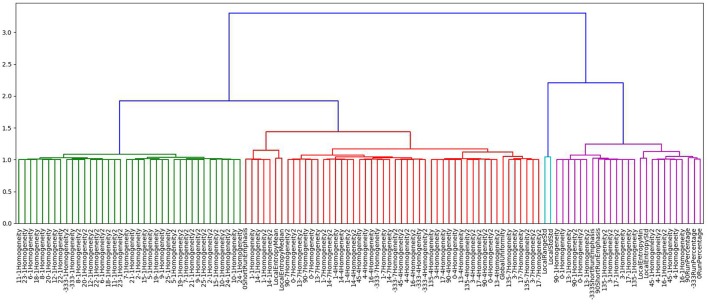
Dendogram generated using agglomerative hierarchal clustering showing just one of the clusters formed based on this method. Depending on the level of the clustering chosen, the number of clusters formed is variable.

The study aimed to find a subset of imaging features most predictive in predicting PSR values. In this analysis, PSR values were used as a continuous variable. To find a subset of features that is relevant in predicting PSR, initially, agglomerative hierarchical clustering was performed based on the Pearson correlation coefficient between features. Hierarchical clustering was selected for being a simple, straightforward clustering measure with easily interpretable results. Agglomerative hierarchical clustering finds clusters by iteratively merging the two most similar features, or groups of features, together. The features with highest correlation coefficients are grouped together first. The results of hierarchical clustering can be presented as a dendogram seen in Figure 1, where the number of clusters depends on what level the dendogram tree is cut. This particular cluster has correctly identified features that fall into the Gray Level Co-occurrence Matrix 3 feature set.

**Figure 2:**
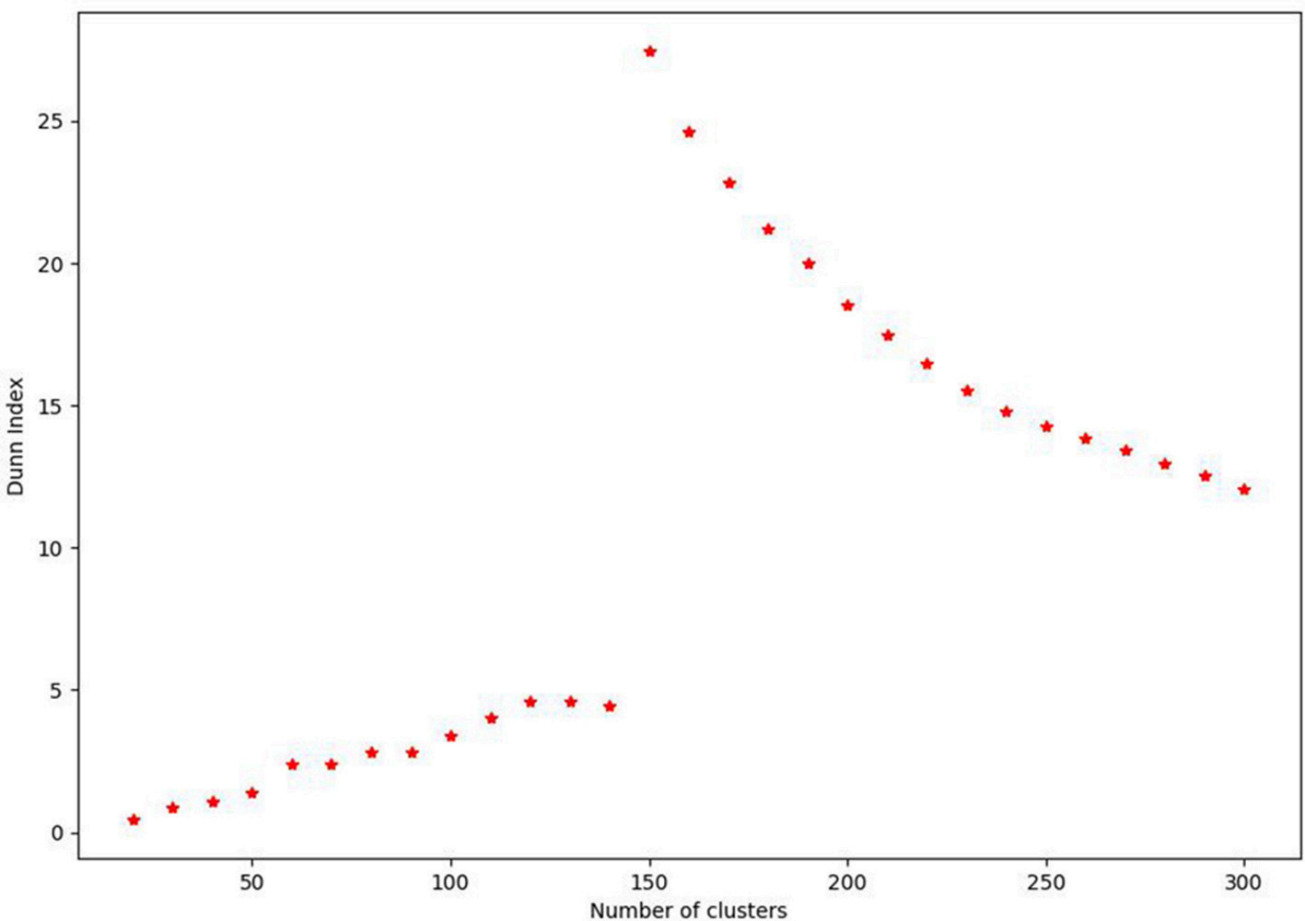
Plot demonstrating calculation of the Dunn Index for the agglomerative hierarchal clustering performed for the radiomic features of this study. From 0 to 144, there is a gradual increase in the Dunn Index before a marked increase at 145 followed by decreasing values. This indicates that 145 clusters are the optimal value for this data set.

The optimal number of clusters was chosen by iteratively decreasing the cluster size and evaluating the Dunn index (30). The Dunn index is defined as the ratio between closest inter-cluster distances and the furthest intra-cluster distance. The largest value of the Dunn index provides the optimal number of partitions in the data. Using this procedure, we found the optimal number of feature clusters to be 145. This is demonstrated in Figure 2.

Among each cluster set we chose the feature that is least correlated to the rest of clusters. This allows for the reduction of noise and removal of redundancy. In the next step, we use target values of PSR and perform linear regression with squared regularization term. For the penalty term, we chose an intermediate value of 0.6 to avoid over-fitting. Feature ranking was performed based on the normalized absolute values of the weights obtained by linear regression.

### Validation studies

Additionally, validation studies for the results were conducted to ensure that this algorithm was shown to be able discover features that are predictive of tracking. Four categories of feature groups were assessed: (1) features selected by agglomerative hierarchical clustering, (2) all 2,287 features together (3) top four features from the subset of agglomerative clustering, and (4) top nine features solely selected based on Pearson correlation. For each category the data was randomly split into 50% training and 50% testing samples. Ridge regression was done to perform linear fit on the training sample and predict the PSR of the testing sample. For each split, the r-square score was computed for the testing sample and it was repeated five times. The mean r-square of five splits was compared for three categories. The r-square can range between 1 to arbitrary negative values; 1 signifies the features are able to generalize to the dataset and negative values imply the model can be arbitrarily worse.

## Results

**Figure 3:**
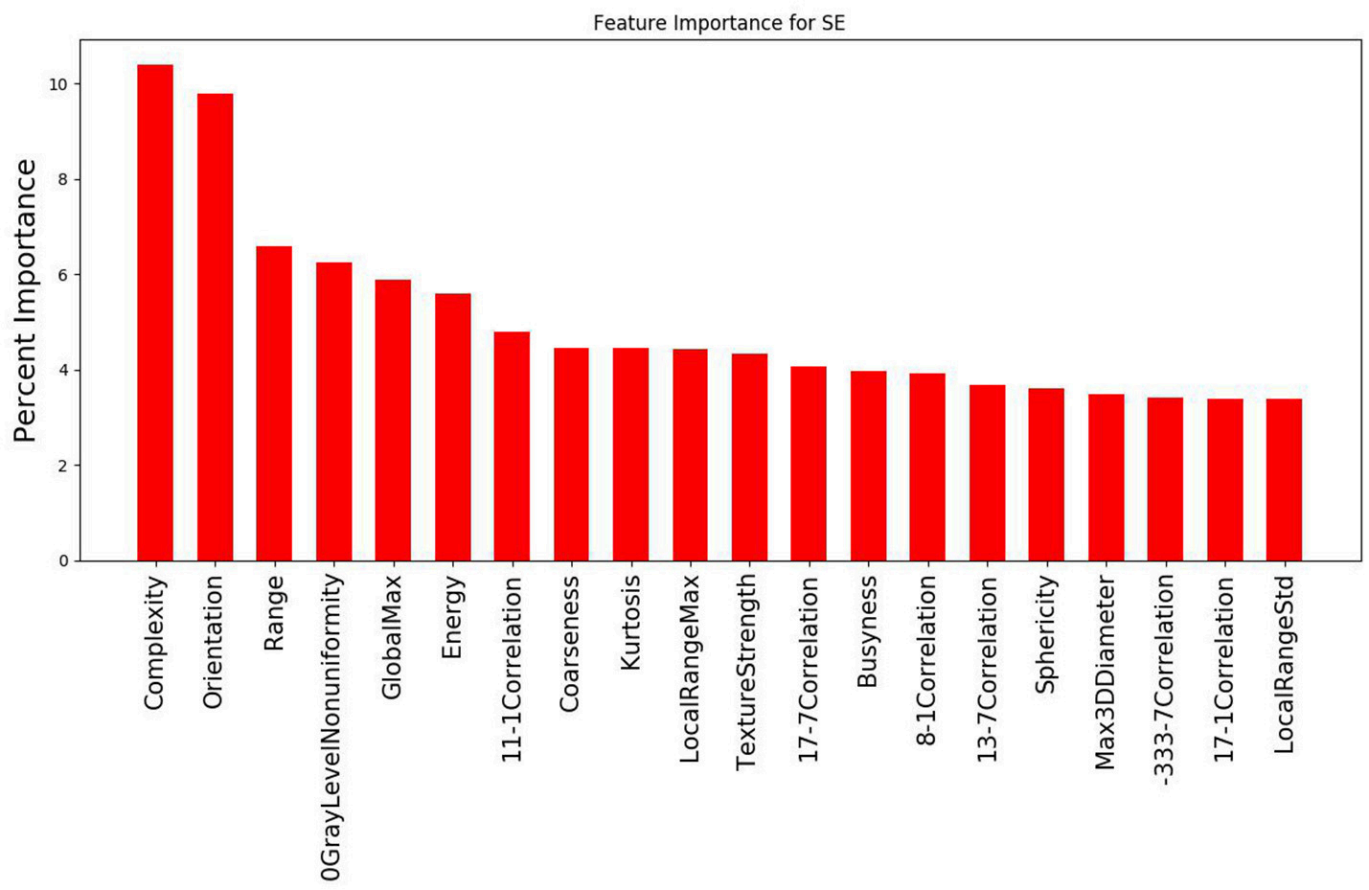
Graph demonstrating the 20 features with the strongest relationship to PSR for the SE imaging data out of the 145 cluster sets. Complexity and orientation were the most predictive of PSR for SE.

The study evaluated 1,419 radiomic features for 8 patients which was narrowed down using agglomerative hierarchal clustering to 145 clusters. From there, the 20 features with the strongest relationship to the PSR value are summarized in Figure 3, 4. In the analysis of the radiomics features, it was found that there were several features that showed high predictive potential for PSR. The features complexity, orientation, gray level non-uniformity and range demonstrated the highest degree of correlation with PSR for both SE and DE imaging. Other radiomic features with high correlation coefficients are also included as well. Of note, many of the intensity based features demonstrated high correlation, including: energy, globalmax, kurtosis, localrangemax, and localrangestd. SE and DE demonstrated similar results in regards to correlative features. Of all the radiomic features highlighted, all *p*-values were < 0.05 indicating statistical significance of the correlation of the feature to the SE and DE PSR values.

**Figure 4:**
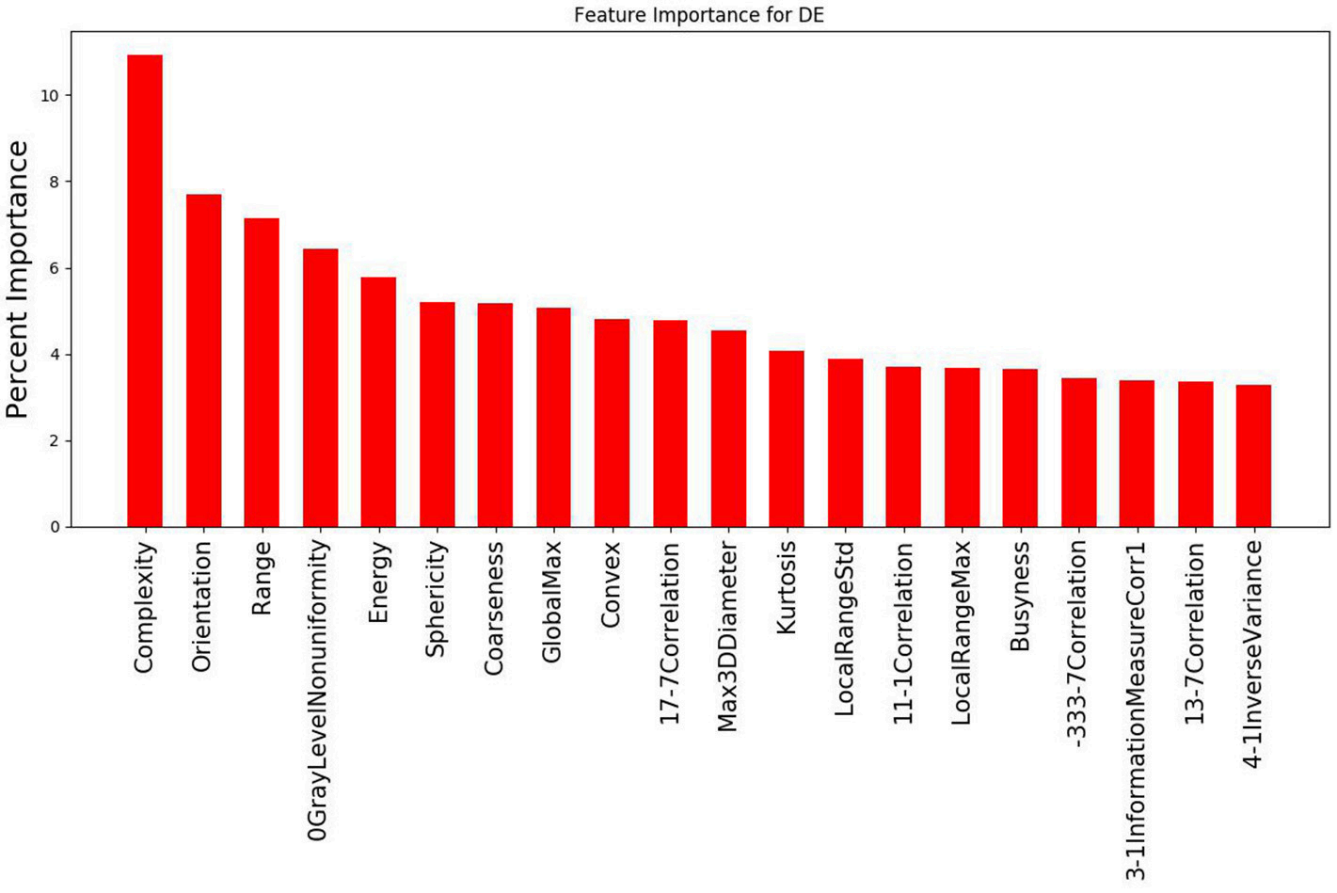
Graph demonstrating the 20 features with the strongest relationship to PSR for the DE imaging data out of the 145 cluster sets. Complexity and orientation were the most predictive of PSR for DE, similar to the results for SE.

The validation study revealed that the mean r-square of features in Category 1 (those selected by the method used in the study) were higher than in Category 2, 3, or 4. That is, the features selected by the method in the study are able to generalize the data to a better degree than all 2,287 features together or single features alone. Category 1 had a r-square of 0.29 +/– 0.18 while Category 2 had a r-square of −3.24 +/– 2.32. Category 3, consisting of only the top four features from the selected feature set, had a r-square score −4.13 +/– 8.62. Category 4 consisted of single features and a sampling of the features with highest correlation shows that SurfaceArea, 333GrayLevelNonuniformity and Energy had a r-square of 0.08 +/– 0.36, −0.1 +/−0.63, and −0.14 +/– 0.51, respectively. Based on this validation for the features selected in this study, Category 1 demonstrates better prediction that either the remaining categories.

## Discussion

In this study, we evaluated the correlation between radiomic parameters and the accuracy of a template-based markerless motion tracking algorithm. Overall, a high correlation coefficient was observed between several radiomic features and the PSR values for both SE and DE images. The significance of these results is that PSR >3 have been shown to have improved tracking accuracy. Thus, any features correlated with the PSR value would be able to indicate the potential for quality tracking. In turn, this may allow for the identification of patients which are ideal candidates for using the proposed markerless motion tracking algorithm during SBRT delivery.

For both SE and DE imaging, the top 3 features with the strongest relationship to the PSR were identical: complexity, orientation, and range. Complexity is a feature of the neighbor intensity difference category. Complexity is determined by the amount of primitive components within an image, making it non-uniform due to rapid change in the gray level intensity. Increased complexity can potentially increase the ability to track heterogeneous tumors, which will likely have more complexity compared to normal healthy tissues, which should be more regular. Orientation is a feature of the shape category related to the directionality of the region of interest (ROI), which is reflective of the shape of a tumor. Range is a feature of the intensity direct category that represents the range of gray values in the ROI. This is expected to have a large effect on the ability to track in a similar fashion to complexity. A wider range of gray values will more likely indicate heterogeneity, which serves as a good contrast against more homogenous healthy tissue.

There were several limitations in the scope of this study with the key factor being the small sample size of only 8 patients. Sample size is an important variable is assessing the predictive value of a particular set of radiomic parameters. A small sample size has a larger margin of selection bias, which would mean that the conclusions from this study are not as widely applicable as desired. Zhang et al. (31) demonstrated that a patient cohort of >50 is needed for convergence of the area-under-the curve (AUC) in their analysis. Figueroa et al. (32) predicted a sample size ranging from 80 to 560 is required to reduce the root-mean-square error to <0.01. Moreover, it is not clear how the size of the patient cohort would affect the clustering of radiomic parameters. The next step forward for this study would be applying what was learned here to a larger patient cohort in the aims of corroborating these findings. There are some additional challenges to the process of radiomics as results may differ institutionally or even on different imaging devices. One of the main goals currently in radiomics is standardization of imaging acquisition to ensure consistent and reliable results (33).

This study demonstrated several radiomic features that are positively correlated to PSR, an indicator of how well markerless motion tracking will function for a specific tumor in a patient. Based on the predictive relationship between the PSR value and the radiomic features, ultimately the goal is to use CT imaging data to determine if a patient would make a good candidate for markerless motion tracking SBRT. Radiomics thus offers tremendous potential in improving individualized treatment plans.

## Author contributions

RP, MS, AB, MMH, and JR contributed to the acquisition of this clinical data used in this study. KN, MH, and HM contributed to the data analysis and methodology. KN and MH performed the radiomic analysis and machine learning used in this analysis, as well as drafted the manuscript. All co-authors contributed to the review and editing of the manuscript. JR was the senior author and supervised all aspects of the project.

### Conflict of interest statement

Loyola University has a Master Research Agreement with Varian Medical Systems. The authors declare that the research was conducted in the absence of any commercial or financial relationships that could be construed as a potential conflict of interest.
